# Paralysis of the Facial Nerve

**Published:** 1858-10

**Authors:** 


*Paralysis of the Facial Nerve.*—An interesting case of this disease has been reported by Professor Hohl, of the Lying-in Hospital, at Halle. It occurred in a new-born child that had been removed from the mother by the aid of forceps. The pressure of the left blade upon the side of the head induced a paralysis of the facial nerve which resisted all treatment. In consequence of this, the child was prevented from drawing the milk from the mother's breast, since all the fluid ran out of the corner of the mouth on the paralyzed side. Nothing could be swallowed until it was placed within the grasp of the muscles of the pharynx. The child died, on the twelfth day, of inanition.

